# Establishment of a Predictive Model for the Efficacy of High‐Intensity Focused Ultrasound in the Treatment of Uterine Fibroids

**DOI:** 10.1002/jum.16718

**Published:** 2025-05-27

**Authors:** Huiqing Li, Yanlei Gao, Xiaoyan Zhang, Weili Hou, Yaru Ma, Rui Shi, Peng Ren

**Affiliations:** ^1^ Department of Pathology Shijiazhuang Maternity & Child Healthcare Hospital Shijiazhuang China; ^2^ Department of Radiology Shijiazhuang Maternal and Child Health Hospital Shijiazhuang China; ^3^ Department of Ultrasound Nankai Hospital, Tianjin Hospital of Integrated Traditional Chinese and Western Medicine Tianjin China; ^4^ Maternal Service Center Shijiazhuang Maternal and Child Health Hospital Shijiazhuang China; ^5^ Department of Gynecology Shijiazhuang Maternal and Child Health Hospital Shijiazhuang China; ^6^ First Department of Gynecology Shijiazhuang Maternal and Child Health Hospital Shijiazhuang China; ^7^ Department of Focused Ultrasound Therapy Shijiazhuang Maternal and Child Health Hospital Shijiazhuang China

**Keywords:** high‐intensity focused ultrasound, interaction, nomogram, risk model, uterine fibroids

## Abstract

**Objectives:**

High‐intensity focused ultrasound (HIFU) has demonstrated efficacy as a non‐invasive treatment for uterine fibroids, though individual variability exists. This study aims to develop a risk scoring model using clinical and biochemical features to predict HIFU treatment outcomes.

**Methods:**

This study collected clinical data from patients receiving HIFU treatment, including demographic characteristics, clinical symptoms, treatment information, and biochemical indicators. A risk scoring model was constructed using the random forest analysis method, and its performance was evaluated. Meanwhile, the impact of risk models and other factors on the efficacy of HIFU was evaluated. Furthermore, the interrelationships between the risk model and other factors were explored through interaction analysis. Finally, a nomogram was developed to evaluate its clinical utility.

**Results:**

The risk model, 4 or more treatments, age, and tumor necrosis factor levels were identified as independent influencing factors, with the risk model demonstrating the best performance (area under the curve (AUC) = 0.693). Interaction analysis revealed a significant synergistic effect between the risk model and receiving 4 or more treatments. The nomogram analysis indicated that lower risk scores and fewer treatment sessions were associated with better HIFU treatment outcomes. The receiver operating characteristic curves and calibration curves in both the training and validation sets demonstrated good performance of the nomogram.

**Conclusions:**

This study successfully constructed a risk scoring model based on clinical features and biochemical indicators, which can effectively predict the efficacy of HIFU treatment for uterine fibroids. There is a significant interaction between the risk model and 4 or more treatments. The constructed nomogram provides strong support for individualized treatment.

**Abbreviations**
BMI: body mass index
CRP: C‐reactive protein
FSH: follicle stimulating hormone
HIFU: high‐intensity focused ultrasound
IL‐6: interleukin‐6
MRI: magnetic resonance imaging
NPV: non‐perfused volume
NPVR: NPV ratio
OOB: out‐of‐bag
RF: random forest
ROC: receiver operating characteristic
TNF‐α: tumor necrosis factor alpha

Uterine fibroids are the most common benign tumors in women, typically formed by the proliferation of smooth muscle cells.^1^, ^2^, ^3^ The incidence of uterine fibroids increases with age, particularly among women aged 30–50. About 50% of women may experience uterine fibroids before the age of 50.^4^ Although uterine fibroids are mostly benign, they may lead to symptoms such as heavy menstrual bleeding, abdominal pain, lower back discomfort, and infertility, all of which can markedly reduce a patient's quality of life.^5^ In some cases, larger uterine fibroids may also compress surrounding organs, leading to problems such as difficulty urinating and constipation.^6^ For women with fertility needs, uterine fibroids may affect conception and pregnancy,^7^, ^8^ so personalized treatment decisions need to be made based on the patient's symptoms and specific circumstances.

In recent years, with the progress of society and the advancement of medical technology, the methods for treating uterine fibroids have become increasingly diverse, such as drug therapy,^9^ surgical treatment,^10^ minimally invasive treatment,^11^ etc. High‐intensity focused ultrasound (HIFU), as a non‐invasive therapeutic approach, has gradually emerged as a promising option.^12^, ^13^ Through focusing, HIFU concentrates highly intense ultrasound energy on fibroid tissues to achieve thermal ablation, which results in the reduction of the fibroid volume without resection, avoiding the trauma and recovery period caused by traditional surgery.^14^ Although HIFU has demonstrated substantial therapeutic benefits in many patients, its effectiveness varies significantly among individuals.^15^ This difference is not only closely related to the clinical characteristics, symptoms, and pathological conditions of patients, but may also be influenced by multiple biochemical indicators.^16^, ^17^ Therefore, accurately predicting the efficacy of HIFU in treating uterine fibroids has become a critical challenge in clinical practice.

At present, the prediction of HIFU treatment efficacy is mostly based on imaging data and clinical features.^18^, ^19^ Although these factors provide useful information, they cannot fully reflect the complexity of the patient's hormonal environment. There is currently limited research on using hormone levels (such as Estradiol) to construct risk models for predicting the efficacy of HIFU. Building on this, the study aims to develop a risk scoring model using clinical and biochemical characteristics to predict the effectiveness of HIFU treatment for uterine fibroids. Additionally, it will also explore the impact of the interaction between risk models and different clinical features and treatment factors on efficacy and further validate the clinical applicability of the model through nomogram.

## Materials and Methods

### Research Object

This retrospective cohort study included patients treated for uterine fibroids at our hospital between December 2020 and December 2023. Inclusion criteria include: 1) patients diagnosed with uterine fibroids; 2) patients receiving HIFU treatment; 3) having complete clinical data and biochemical indicators. Exclusion criteria include: 1) pregnant; 2) coexisting malignant tumors; 3) incomplete clinical data. In the end, a total of 340 patients were included in the study.

### Specific Steps of HIFU Treatment

Preoperative magnetic resonance imaging (MRI) is used to confirm the location, volume, blood supply, and relationship of the uterine fibroids with surrounding tissues. Twenty minutes before treatment, Fentanyl and Midazolam are intravenously infused for pain management. The patient lies in a prone position on the treatment bed, with the anterior abdominal wall in contact with degassed water. A catheter is inserted into the bladder and filled with degassed saline to ensure proper bladder filling. The treatment is performed using a scanning method of point‐to‐line, line‐to‐plane, and plane‐to‐body. The ultrasound frequency is set between 0.8 and 4 MHz, with an input power of 100–400 W, and each pulse duration is set between 150 and 1000 ms. Each treatment session lasts between 30 minutes and 2 hours, and the treatment interval is typically 2–4 weeks, depending on the patient's condition. The total treatment duration is 1–3 months.

### Efficacy Evaluation

One month after treatment, we use the non‐perfused volume (NPV) ratio as an efficacy assessment. A ratio below 80% is defined as no improvement, while a ratio greater than 80% is defined as improvement. The specific calculation formula is:

$$\mathrm{NPV}\;\text{ratio (NPVR)}=\text{Non}-\text{perfused volume}\;\left(\mathrm{NPV}\right)/\text{total volume}$$

The NPV refers to the volume with no blood supply observed after treatment, and the total volume refers to the total volume of the uterine fibroid after treatment. Both NPV and total volume are obtained through MRI.

### Data Collection

Collect demographic information of the patients. In the study, MRI was also used to determine the volume (mL), number (single or multiple), and type (solid or mixed) of uterine fibroids. Treatment‐related factors include ultrasound frequency (MHz), ultrasound energy, number of treatments (1, 2–3, or 4 or more), and duration of treatment. Assessing the pre‐treatment clinical symptoms included menorrhagia, dysmenorrhea, compression symptoms, abdominal pain, and anemia, all classified as mild, moderate, or severe. Biochemical data include estradiol (E2, pg/mL) and follicle stimulating hormone (FSH, mIU/mL) during the follicular phase, C‐reactive protein (CRP, mg/L), tumor necrosis factor alpha (TNF‐α, pg/mL), and interleukin‐6 (IL‐6, pg/mL). These data were collected before surgery.

### Statistical Analysis

Random forest (RF) analysis is used to select the best features, and the best “mtry” value is determined using the out‐of‐bag (OOB) error estimate. Based on the determined best value, a random forest model is constructed, and the model performance is evaluated using receiver operating characteristic (ROC) curve, accuracy, precision, recall, and F1 score. The GINI index in the random forest is used as the feature importance, and the top 5 features with the highest GINI index are selected to construct a risk model. The specific formula is as follows:

$$\text{Risk model}=\begin{array}{l} {\text{Factor}}_{\left[1\right]}\times {\text{GINI}}_{\left[1\right]}+{\text{Factor}}_{\left[2\right]}\\ \times {\text{GINI}}_{\left[2\right]}+{\text{Factor}}_{\left[3\right]}\times {\text{GINI}}_{\left[3\right]}\\ +\ldots {\text{Factor}}_{\left[n\right]}\times {\text{GINI}}_{\left[n\right]} \end{array}$$

Using multiple logistic regression analysis to examine the impact of the risk model and its interactions on therapeutic efficacy, the dataset was divided into a training set and a test set in a 7:3 ratio. The risk model and significant factors in the multiple logistic regression model were constructed into a nomogram in the training and test sets. Continuous data are presented as median (range) and analyzed using the Mann–Whitney U test or t‐test, while categorical data are presented as frequency (percentage) and analyzed using chi‐square or Fisher's exact test.

## Results

### Differences in Demographic and Clinical Characteristics, as Well as Treatment‐Related Information Between the Improved and Unimproved Groups

In this study, 85% of patients showed improvement, while 15% did not. There are significant differences between the improvement group and the no improvement group in multiple demographic and clinical characteristics, as well as treatment‐related information. The median age, body mass index (BMI), and uterine fibroid volume of the improved group were significantly lower than those of the unimproved group (*P* values were .0417, .019, and .0142, respectively). In addition, the improvement group had a higher proportion of receiving 2–3 treatments in terms of treatment frequency, while the no improvement group had a higher proportion of receiving 4 or more treatments (*P* = .0012). In terms of pre‐treatment clinical symptoms, patients in the improvement group had milder symptoms of menstruation and abdominal pain, while those in the no improvement group showed more severe symptoms (*P* = .038 and .0326, respectively). In terms of biochemical indicators, the estrogen level in the improved group was significantly lower than that in the unimproved group (*P* = .0283), while the CRP and TNF‐α levels in the unimproved group were significantly higher (*P* = .00188 and .0116, respectively). Other factors such as pregnancy history, ultrasound frequency, and ultrasound energy did not show significant differences between the 2 groups (Table 1).

**Table 1:** Improvement and Non‐Improvement Patients' Differences in Demographic Characteristics, Clinical Features, and Treatment‐Related Information

	All Patients (n = 340)	Improvement (n = 289)	No Improvement (n = 51)	P‐Value
Age	39 (27–51)	37 (27–51)	43 (27–51)	.0417
Body mass index, BMI	26.1 (18.9–33.5)	25.0 (19.1–32.8)	27.3 (18.9–33.5)	.019
Pregnancy history				.1773
Yes	258 (75.88%)	215 (74.39%)	43 (84.31%)	
No	82 (24.12%)	74 (25.61%)	8 (15.69%)	
Family history of uterine fibroids				.3729
Yes	43 (12.65%)	39 (13.49%)	4 (7.84%)	
No	297 (87.35%)	250 (86.51%)	47 (92.16%)	
Volume of the fibroid (mL)	53.7 (24.1–75.8)	49.0 (24.1–75.8)	60.2 (25.4–75.5)	.0142
Fibroid number				.1531
Single fibroid	127 (37.35%)	113 (39.1%)	14 (27.45%)	
Multiple fibroids	213 (62.65%)	176 (60.9%)	37 (72.55%)	
Fibroid type				.0513
Solid fibroid	248 (72.94%)	217 (75.09%)	31 (60.78%)	
Mixed fibroid	92 (27.06%)	72 (24.91%)	20 (39.22%)	
Ultrasound frequency (MHz)	2.5 (1.3–3.7)	2.5 (1.3–3.7)	2.5 (1.3–3.7)	.732
Ultrasound energy	899.0 (570.2–1226.9)	895.4 (570.2–1226.9)	908.3 (594.0–1220.6)	.953
Number of treatments				.0012
1	57 (16.76%)	47 (16.26%)	10 (19.61%)	
2, 3	261 (76.76%)	229 (79.24%)	32 (62.75%)	
4 or more	22 (6.47%)	13 (4.5%)	9 (17.65%)	
Treatment duration	60 (35–84)	60 (35–84)	63 (35–84)	.744
Menorrhagia				.0381
Mild	150 (44.12%)	127 (43.94%)	23 (45.1%)	
Moderate	160 (47.06%)	141 (48.79%)	19 (37.25%)	
Severe	30 (8.82%)	21 (7.27%)	9 (17.65%)	
Dysmenorrhea				.2048
Mild	242 (71.18%)	211 (73.01%)	31 (60.78%)	
Moderate	84 (24.71%)	67 (23.18%)	17 (33.33%)	
Severe	14 (4.12%)	11 (3.81%)	3 (5.88%)	
Compression symptoms				.2549
Mild	263 (77.35%)	219 (75.78%)	44 (86.27%)	
Moderate	67 (19.71%)	61 (21.11%)	6 (11.76%)	
Severe	10 (2.94%)	9 (3.11%)	1 (1.96%)	
Abdominal pain				.0326
Mild	176 (51.76%)	148 (51.21%)	28 (54.9%)	
Moderate	151 (44.41%)	133 (46.02%)	18 (35.29%)	
Severe	13 (3.82%)	8 (2.77%)	5 (9.8%)	
Anemia				.2252
Mild	158 (46.47%)	129 (44.64%)	29 (56.86%)	
Moderate	167 (49.12%)	146 (50.52%)	21 (41.18%)	
Severe	15 (4.41%)	14 (4.84%)	1 (1.96%)	
Estradiol, E2 (pg/mL)	226.3 (153.2–293.8)	222.2 (153.2–293.8)	243.8 (254.3–289.0)	.0283
Follicle Stimulating Hormone, FSH (mIU/mL)	10.0 (5.5–14.3)	10.0 (5.5–14.3)	10.1 (5.7–14.3)	.394
C‐reactive protein, CRP (mg/L)	5.4 (2.3–8.7)	5.2 (2.3–8.7)	6.7 (2.3–8.7)	.0019
Tumor Necrosis Factor‐alpha, TNF‐α (pg/mL)	9.1 (6.6–11.5)	9.0 (6.6–11.5)	9.8 (6.7–11.5)	.0116
Interleukin‐6, IL‐6 (pg/mL)	6.1 (4.1–7.9)	5.9 (4.1–7.9)	6.2 (4.4–7.9)	.0101

### Using Random Forest Model to Build Risk Model

**Figure 1:**
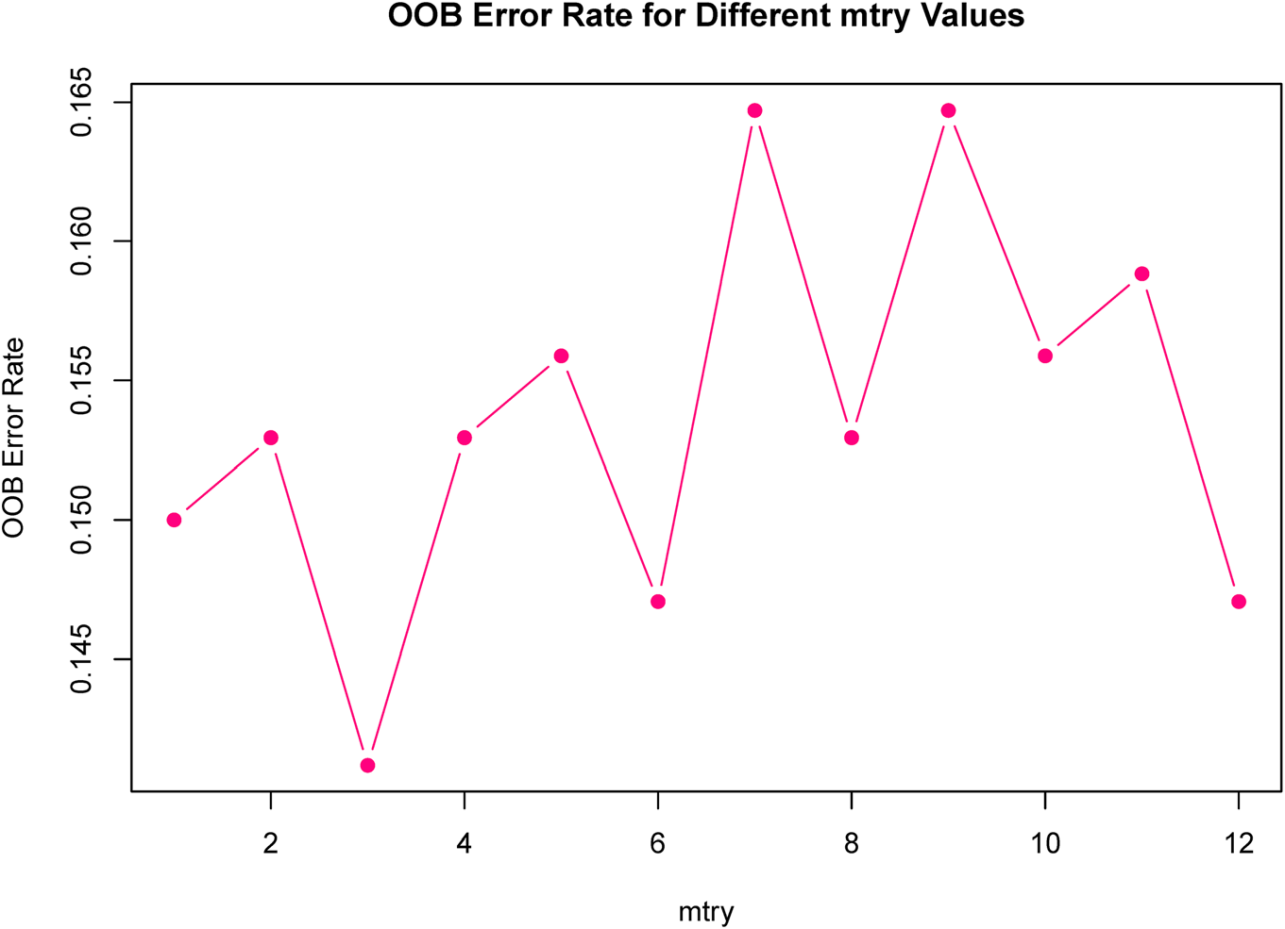
The “mtry” value selected through the OOB error method.

**Figure 2:**
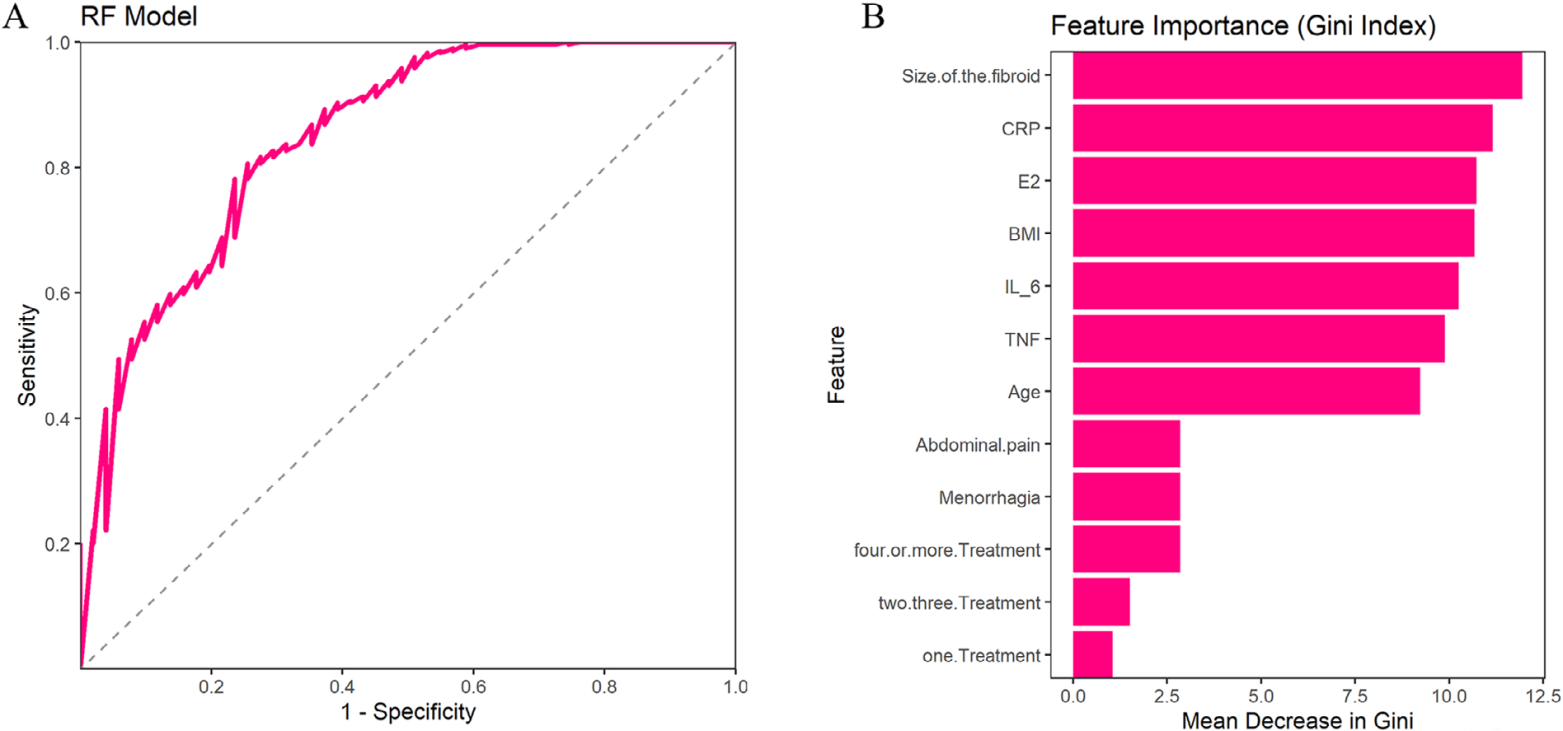
**A**, ROC curve of the random forest model. **B**, GINI index of the random forest model.

We used the above difference indicators as input variables for random forest analysis, and the optimal “mtry” value was 3 (Figure 1), area under the curve (AUC) value of 0.849, indicating that the overall performance of the random forest model was good (Figure 2A). The AUC is 0.849, indicating that the model has good performance in distinguishing positive and negative classes. The sensitivity and specificity are relatively balanced, with a high F1 score and excellent precision, indicating that the model can recognize positive classes well with fewer false positives (Table 2). The visualization results indicate that the top 5 features with the highest GINI index are volume of the fibroid, CRP, E2, BMI, and IL‐6 (Figure 2B).

**Table 2:** ROC Curve Parameters

	AUC	AUC‐CI‐Lower	AUC‐CI‐Upper	Best‐Threshold	Youden	Sensitivity	Specificity	F1 Score	Accuracy	Recall	Precision
RF	0.849	0.788	0.909	0.488	0.551	0.806	0.745	85.606	77.647	78.201	94.561
Risk score	0.693	0.606	0.761	3756.805	0.316	0.747	0.659				
Size of the fibroid	0.606	0.522	0.691	63.865	0.219	0.768	0.451				
CRP	0.636	0.553	0.720	5.929	0.279	0.612	0.667				
E2	0.588	0.502	0.673	228.079	0.197	0.550	0.647				
BMI	0.608	0.526	0.689	27.831	0.210	0.602	0.608				
IL‐6	0.623	0.541	0.705	5.897	0.224	0.498	0.725				
Training set	0.734	0.650	0.818	0.845	0.384	0.690	0.694				
Test set	0.814	0.690	0.938	0.838	0.576	0.753	0.824				

### Construction of Risk Model

**Figure 3:**
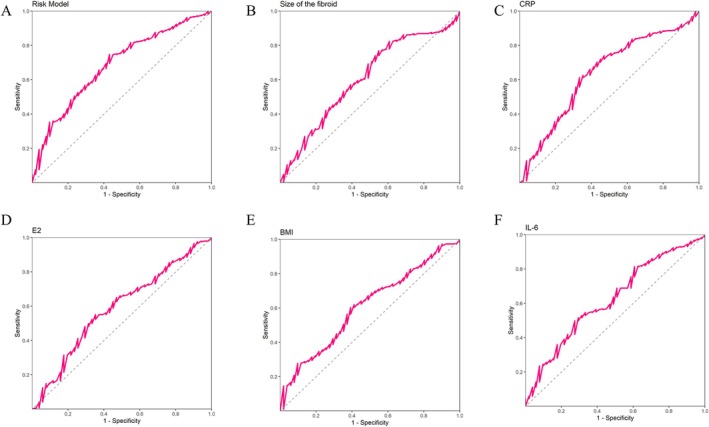
**A**, ROC curve of risk model predicting efficacy. **B**, ROC curve for predicting therapeutic efficacy based on the volume of the fibroid. **C**, ROC curve for predicting therapeutic efficacy using CRP. **D**, ROC curve for predicting therapeutic efficacy using E2. **E**, ROC curve for predicting therapeutic efficacy based on BMI. **F**, ROC curve for predicting therapeutic efficacy of IL‐6.

Evaluate the predictive ability of the risk model and its 5 features for HIFU treatment efficacy. The results showed that the risk model had the highest AUC value of 0.693, indicating that it performed well in distinguishing treatment effects. Its optimal threshold was 3756.805, sensitivity was 0.747, and specificity was 0.569. Other indicators such as volume of the fibroid (AUC = 0.606) and CRP (AUC = 0.636) also showed certain predictive ability, especially with high sensitivity of CRP (0.612). In addition, the AUC values of estrogen (E2), BMI, and IL‐6 were 0.588, 0.608, and 0.623, respectively, indicating that these factors also have certain reference value in predicting the efficacy of HIFU. Overall, the risk model performed the best among all indicators (Table 2; Figure 3).

### Multivariate Logistic Regression Analysis of Risk Models and Their Interaction Effects for Predicting Therapeutic Efficacy

A multivariate logistic regression analysis was performed using the top 10 features ranked by the Gini index in Figure 2B, along with the risk model. The results showed that patients who received 4 or more treatments had a lower likelihood of improvement compared to those who received less treatment (OR = 0.763, *P* < .001), indicating that the more treatments, the lower the probability of improvement. There is also a significant negative correlation between age and the likelihood of improvement (odds ratio (OR) = 0.995, *P* = .047), meaning that the older the age, the lower the likelihood of improvement in treatment efficacy. The level of TNF also has a significant impact on treatment efficacy, and as the level of TNF increases, the likelihood of patient improvement slightly decreases (OR = 0.974, *P* = .048). There is also a significant negative correlation between the risk model and the improvement effect (OR = 0.710, *P* = .005), indicating that the higher the risk model score, the worse the therapeutic effect. Other variables, such as excessive menstruation and abdominal pain, did not show a significant impact on improvement (*P* > .05) (Table 3).

**Table 3:** Analyzing the impact of risk models and their interactions on the efficacy of HIFU

	Estimate	Std. Error	Statistic	P‐Value	OR	CI_lower	CI_upper
Four or more treatment	−0.270	0.076	−3.539	.000	0.763	0.657	0.887
Age	−0.005	0.003	−1.990	.047	0.995	0.989	1.000
Menorrhagia	−0.017	0.030	−0.589	.556	0.983	0.928	1.041
Abdominal pain	−0.019	0.033	−0.584	.560	0.981	0.920	1.046
TNF	−0.026	0.013	−1.986	.048	0.974	0.949	1.000
Risk model	−0.342	0.122	−2.803	.005	0.710	0.560	0.900

	Estimate	Std. Error	Statistic	P‐Value	OR	CI_lower	CI_upper
Risk model	0.000	0.000	−0.935	.351	1.000	0.999	1.000
Age	−0.014	0.021	−0.654	.514	0.986	0.946	1.028
Risk model × age	0.000	0.000	0.395	.693	1.000	1.000	1.000

	Estimate	Std. Error	Statistic	P‐Value	OR	CI_lower	CI_upper
Risk model	−0.001	0.000	−1.862	.063	0.999	0.999	1.000
TNF	−0.171	0.104	−1.648	.100	0.843	0.688	1.033
Risk model × TNF	0.000	0.000	1.367	.173	1.000	1.000	1.000

	Estimate	Std. Error	Statistic	P‐Value	OR	CI_lower	CI_upper
Risk model	0.000	0.000	−3.719	.000	1.000	1.000	1.000
Four or more treatment	−1.518	0.624	−2.431	.016	0.219	0.064	0.745
Risk model × 4 or more treatment	−0.832	0.363	−2.294	.022	0.436	0.186	0.701

From this, risk model, age, TNF, and 4 or more treatments are risk factors for improvement. Continuing with the analysis of the interaction between risk models and other risk factors, the results showed that there was only a significant interaction with 4 or more treatments, and the B value was less than 0, indicating that the risk model was more effective in patients treated with 4 or more treatments, and the B value was smaller than that of the risk model alone, indicating that 4 or more treatments were factors that strengthened the predictive effect of the risk model on efficacy. The insignificant interaction between age and TNF indicates that the risk model is not limited by age and TNF levels (Table 3).

### Building Nomogram

**Figure 4:**
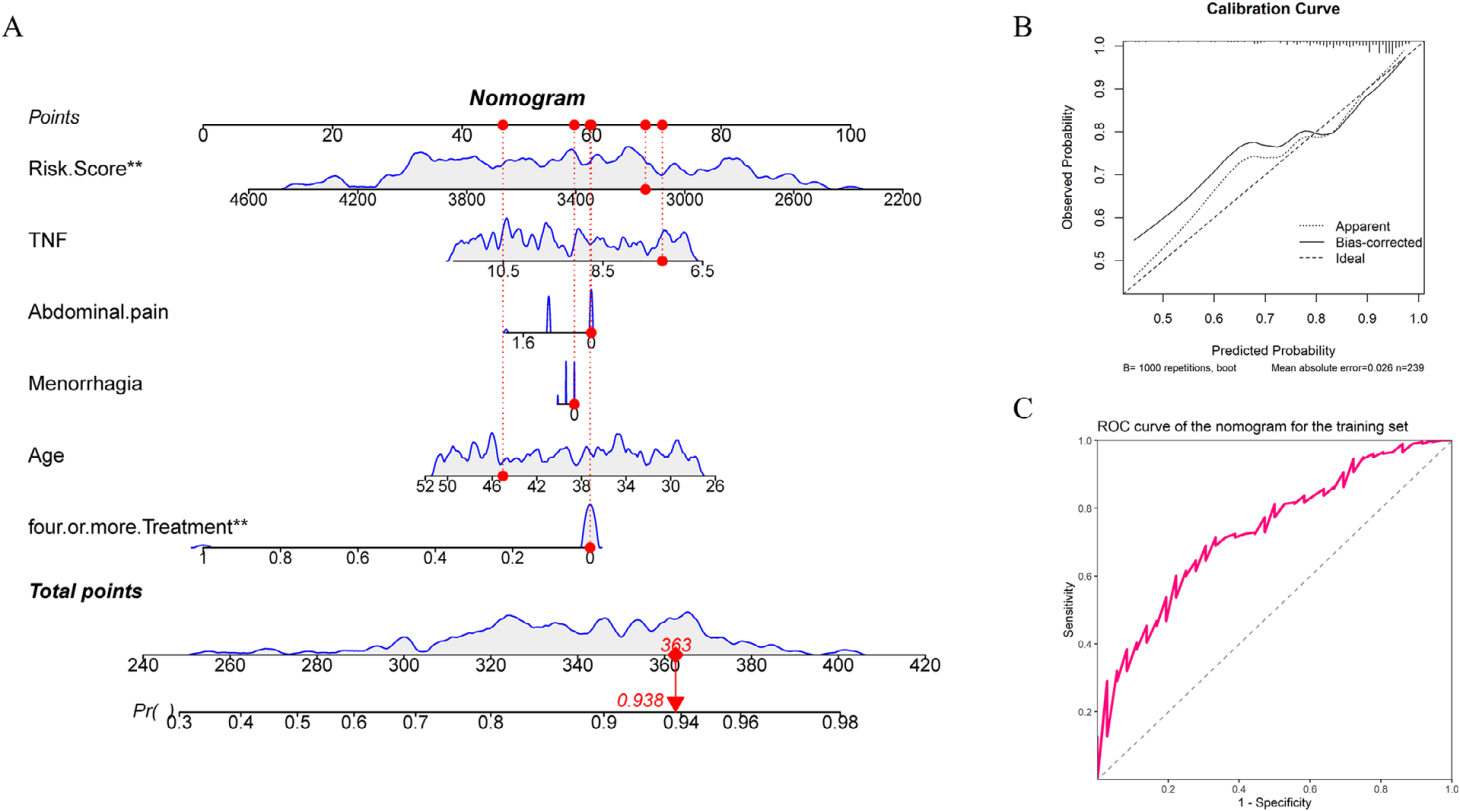
**A**, Nomogram of the training set. **B**, Calibration curve of the training set nomogram. **C**, ROC curve of the training set nomogram.

**Figure 5:**
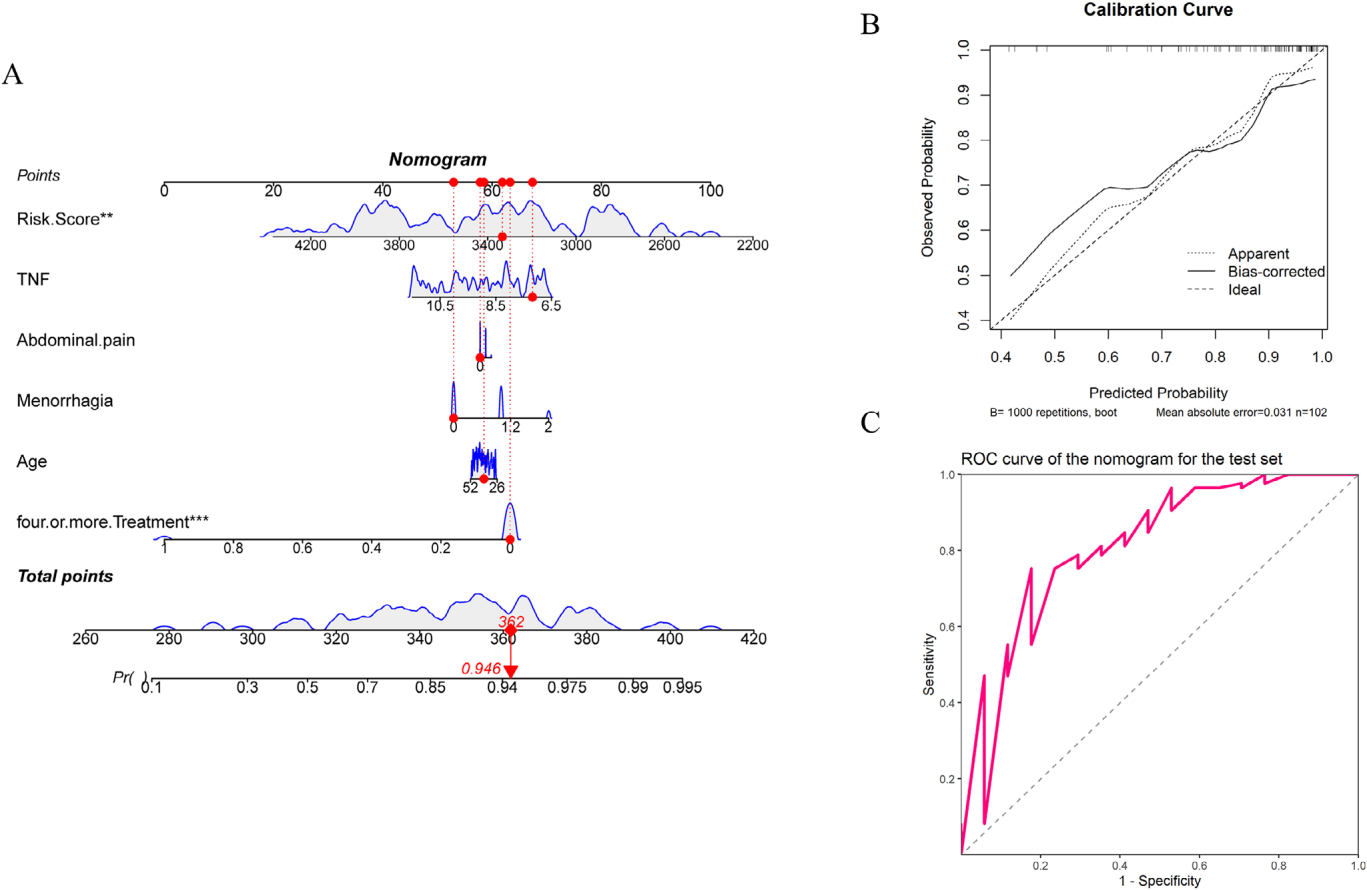
**A**, Nomogram of the test set. **B**, Calibration curve of the test set nomogram. **C**, ROC curve of the test set nomogram.

The results show that in the training set, the risk model is significantly higher than that of 4 or more treatments. The lower the risk model and the fewer treatments, the better the final efficacy. According to the clinical data of the patient marked in the figure, the risk model is 3144, TNF level is 7.308, mild abdominal pain symptoms, mild menorrhagia symptoms, age is 45 years treatment frequency is less than 4 times, total score is 363, and the probability of improvement in HIFU treatment effect is 93.8%. The AUC value of the nomogram model is 0.734, indicating that the model has high prediction accuracy, and the calibration curve is close to the diagonal, indicating that the model has good calibration in the prediction process (Table 2) (Figure 4). In the test set, the same risk model and 4 or more treatments were significant. The lower the risk model and the fewer treatments, the better the therapeutic effect. According to the clinical data of the patient marked in the figure, the risk model is 3333, TNF level is 7.182, mild abdominal pain symptoms, mild menorrhagia symptoms, age is 39 years treatment frequency is less than 4 times, total score is 362, and the probability of improvement with HIFU treatment is 94.6%. The AUC value of the nomogram model is 0.814, which has high accuracy. The calibration curve falls near the diagonal, indicating that the results are reliable (Table 2; Figure 5).

## Discussion

This study first utilized a random forest model for feature screening and risk model construction. Secondly, the impact of the risk model and its interaction on the efficacy of HIFU was studied. Finally, a nomogram model was constructed using the risk model and factors that significantly affect the efficacy.

Our risk model is constructed based on 5 indicators: Volume of the fibroid, CRP, E2, BMI, and IL‐6. CRP and IL‐6 are inflammatory factors, while estradiol is one of the most important estrogens in the female body.^20^ Currently, most research is based on radiomics data to build predictive models. The innovation of our risk model lies in the use of hormone levels. Research has found that high levels of estradiol are closely related to the occurrence, volume, and growth of uterine fibroids.^21^, ^22^ There is a complex interaction between hormones and inflammatory factors in the occurrence and progression of uterine fibroids. Our model can accurately evaluate the efficacy of HIFU by combining hormone levels with inflammatory factors.

Our study found that the risk model performed better than other indicators, suggesting its higher accuracy and clinical utility. Other features, such as estradiol (E2), BMI, and IL‐6, also demonstrated some predictive value for HIFU treatment efficacy. The risk score incorporates multiple clinical, demographic, and biomarker factors, making it more comprehensive and better able to reflect patients' overall condition. This model can effectively identify patients with improved treatment outcomes (high sensitivity), which helps clinicians stratify patients before treatment. However, its relatively low specificity may lead to some non‐responders being misclassified as responders, which should be carefully considered in clinical practice.

There was a significant negative correlation between age, TNF levels, and treatment efficacy. This may be because as age increases, changes in physiological and biochemical functions may affect the body's response to treatment.^23^ For example, as age increases, the body's ability to repair and regenerate tissues decreases; especially during the treatment process, damaged tissues may not be effectively repaired.^24^ This may lead to a prolonged recovery period after treatment, affecting the improvement of therapeutic efficacy. TNF (tumor necrosis factor‐α) is an important inflammatory factor, and elevated levels of TNF are often associated with chronic inflammation, immune response, and the deterioration of some diseases.^25^ High levels of TNF before treatment are associated with a persistent chronic inflammatory response in the body, which may affect treatment efficacy, especially for local treatments such as HIFU.^26^ Inflammatory reactions may lead to tissue damage, delayed recovery, or incomplete repair in the treatment area, thereby affecting the effectiveness of treatment.

The frequency of treatment is an important finding in this study. The proportion of patients in the improvement group receiving less than 4 treatments is higher, while patients in the no improvement group receive more than 4 treatments. Through multiple logistic regression analysis, the results showed that patients who received 4 or more treatments had a significantly reduced probability of improvement (OR = 0.763, *P* < .001). This finding suggests that an increase in the number of treatments during HIFU therapy may lead to a decrease in treatment effectiveness, which may be related to tissue reactions or accumulated side effects after treatment. Although multiple treatments may increase the cumulative effect of therapeutic efficacy, excessive treatment may trigger excessive tissue reactions, thereby affecting the treatment effect.

Another highlight of this study is that we found that 4 or more treatments to some extent enhanced the predictive ability of the risk model for treatment efficacy. This may be because in patients who received multiple treatments, the effectiveness of treatment may be affected by cumulative effects, treatment tolerance, and side effects, which enhance the predictive ability of the risk model in these patients. Patients who receive more treatments usually undergo a longer treatment process, and there may be a certain level of inflammation in their bodies. Additionally, due to the presence of inflammatory factors (CRP and IL‐6) in our model, risk models can better reflect their treatment efficacy.

When further validating the performance of the risk model in the nomogram, the results of both the training and test sets showed that the risk model and 4 or more treatments were important factors affecting treatment effectiveness. In both the training and test sets, patients with lower risk models and fewer than 4 treatments have a higher probability of treatment improvement, and the nomogram model has high accuracy and good calibration, providing doctors with a quantitative tool to support personalized treatment decisions.

This study has several limitations. First, it is a single‐center, retrospective study, which may lead to a limited sample size and potential selection bias. Second, we only predicted the efficacy of HIFU 1 month after treatment. In the future, large‐scale prospective randomized controlled trials and long‐term efficacy evaluations should be conducted.

## Conclusion

Overall, this study successfully constructed a risk scoring model based on clinical features and biochemical indicators for predicting the efficacy of HIFU treatment for uterine fibroids. Risk model, number of treatments, age, and TNF level are independent risk factors for treatment efficacy. Interaction analysis shows a significant synergistic effect between risk model and 4 or more treatments. The AUC values of the nomogram are 0.734 and 0.814, indicating high predictive accuracy and good calibration, providing strong support for personalized treatment.

## Ethics Statement

This paper has been reviewed by relevant departments of our hospital, such as the Science and Education Department, Medical Department and Ethics Committee of Shijiazhuang Maternity & Child Healthcare Hospital, Shijiazhuang. The research content involved in this research meets the requirements of medical ethics and academic morality of our hospital, and the research content is reasonable, the risks are controllable, and there are no violations. The relevant research carried out is in line with the safe, standardized and true scientific research guiding principles, and in line with the requirements of the clinical research ethics code.

## Data Availability

The data that support the findings of this study are available on request from the corresponding author. The data are not publicly available due to privacy or ethical restrictions.
